# Motor problems in children with neurofibromatosis type 1

**DOI:** 10.1186/s11689-017-9198-5

**Published:** 2017-05-19

**Authors:** André B. Rietman, Rianne Oostenbrink, Sanne Bongers, Eddy Gaukema, Sandra van Abeelen, Jos G. Hendriksen, Caspar W. N. Looman, Pieter F. A. de Nijs, Marie-Claire de Wit

**Affiliations:** 1 Department of Child and Adolescent Psychiatry/Psychology, ENCORE NF1 Expertise Centre for Neurodevelopmental Disorders, Erasmus Medical Centre–Sophia Children’s Hospital, Rotterdam, The Netherlands; 2 Department of General Paediatrics, ENCORE NF1, Erasmus Medical Centre–Sophia Children’s Hospital, Rotterdam, The Netherlands; 3 Department of Paediatric Neurology, ENCORE NF1, Erasmus Medical Centre–Sophia Children’s Hospital, Rotterdam, The Netherlands; 4 Department of Public Health, Erasmus Medical Centre, Rotterdam, The Netherlands; 5 Kempenhaeghe Centre for neurological learning disabilities, Heeze, The Netherlands; 6 Department of Child and Adolescent Psychiatry/Psychology, Sophia Children’s Hospital, Room Sp 2478, P.O. Box 2060, 3000 CB Rotterdam, The Netherlands

**Keywords:** Neurofibromatosis type 1, Motor problems, DCD, Emotional and behavioural problems, Intelligence

## Abstract

**Background:**

Children with the neurogenetic disorder neurofibromatosis type 1 (NF1) often have problems with learning and behaviour. In both parent reports and neuropsychological assessment, motor problems are reported in approximately one third to one half of the children with NF1. Studies using broad motor performance test batteries with relatively large groups of children with NF1 are limited. The aim of this cross-sectional observational study was to describe the severity of motor problems in children with NF1 and to explore the predictive value of demographics, intelligence, and behavioural problems.

**Methods:**

From 2002 to 2014, 69 children with NF1, aged 4 to 16 years (age = 9.5 ± 2.8 years; 29 girls) had a motor, psychological, and neurological evaluation in an NF1 expertise centre. Data were collected about (1) motor performance (M-ABC: Movement Assessment Battery for Children), (2) intelligence, and (3) emotional and behavioural problems as rated by parents.

**Results:**

Sixty-one percent of these children scored within the clinical range of the M-ABC. In ordinal logistic regression analyses, motor problems were associated with symptoms of attention-deficit/hyperactivity disorder (ADHD), symptoms of autism spectrum disorder (ASD), and externalising behavioural problems. Motor outcome was not predicted by age, intelligence, scoliosis, hypotonia, nor hypermobility.

**Conclusions:**

Motor problems are among the most common comorbid developmental problems in children with NF1, and these problems do not diminish with age. Because of their impact on daily functioning, motor problems need to be specifically addressed in diagnosis, follow-up, and treatment of NF1.

## Background

Neurofibromatosis type 1 (NF1) is an autosomal dominant neurogenetic disorder with an incidence of at least 1:2700 [1]. Although NF1 is defined by cutaneous and neurological symptoms such as café-au-lait spots and neurofibromas, the most common complications in childhood are deficits of cognition and of social and emotional development [2]. The prevalence of neuropsychiatric problems such as attention-deficit/hyperactivity disorder (ADHD) and autism spectrum disorder (ASD) is much larger than in the general population [3]. In both parent reports and neuropsychological assessments, motor problems are reported in approximately one third to one half of the children with NF1 [4, 5]. Almost 30% of children with NF1 had received occupational therapy [6], and over 40% receive remedial teaching for motor problems at school [7]. NF1-related skeletal and muscular abnormalities, such as scoliosis, pseudo-arthrosis, decreased bone strength, and reduced muscle strength may be associated with motor problems in NF1 [5]. Motor problems can hinder a child’s participation at school and in play, sports, and peer-group activities, but they may also affect social and emotional development [8]. In our expertise centres for NF1, motor problems are among the most common complaints, which is the reason for the structural assessment of motor skills presented in this study.

Previous studies on motor skills in NF1 have often used selective tests, targeting only parts of the motor domain [2, 9]. Studies using a small selection of motor or constructional tests do not show the full range of motor problems in children with NF1. Broader test batteries for both fine and gross motor skills have been used in a limited number of smaller studies [5, 10] or when focusing on young children [4, 11]. Recently, [9] a broad test battery (the BOT-2) was used with 46 children, from 7 to 17 years old, to establish correlations between problems in motor and cognitive domains. In this study, cognition was associated with balance, gait, running speed, and agility in children with NF1. A shared abnormal neurodevelopmental process underlying cognitive and motor abilities in NF1 was hypothesised [9].

A study on a large group of children and adolescents with NF1, using a broad test battery for motor performance, could inform health care professionals not only about the association between motor problems and cognitive development but also about the association with the emotional and behavioural problems often present in NF1. Our cross-sectional study aims to describe the presence and severity of motor problems in children and adolescents with NF1 and to explore the associations between these motor problems and background variables, intelligence, and emotional and behavioural problems.

## Methods

### Procedure and patients

**Fig. 1:**
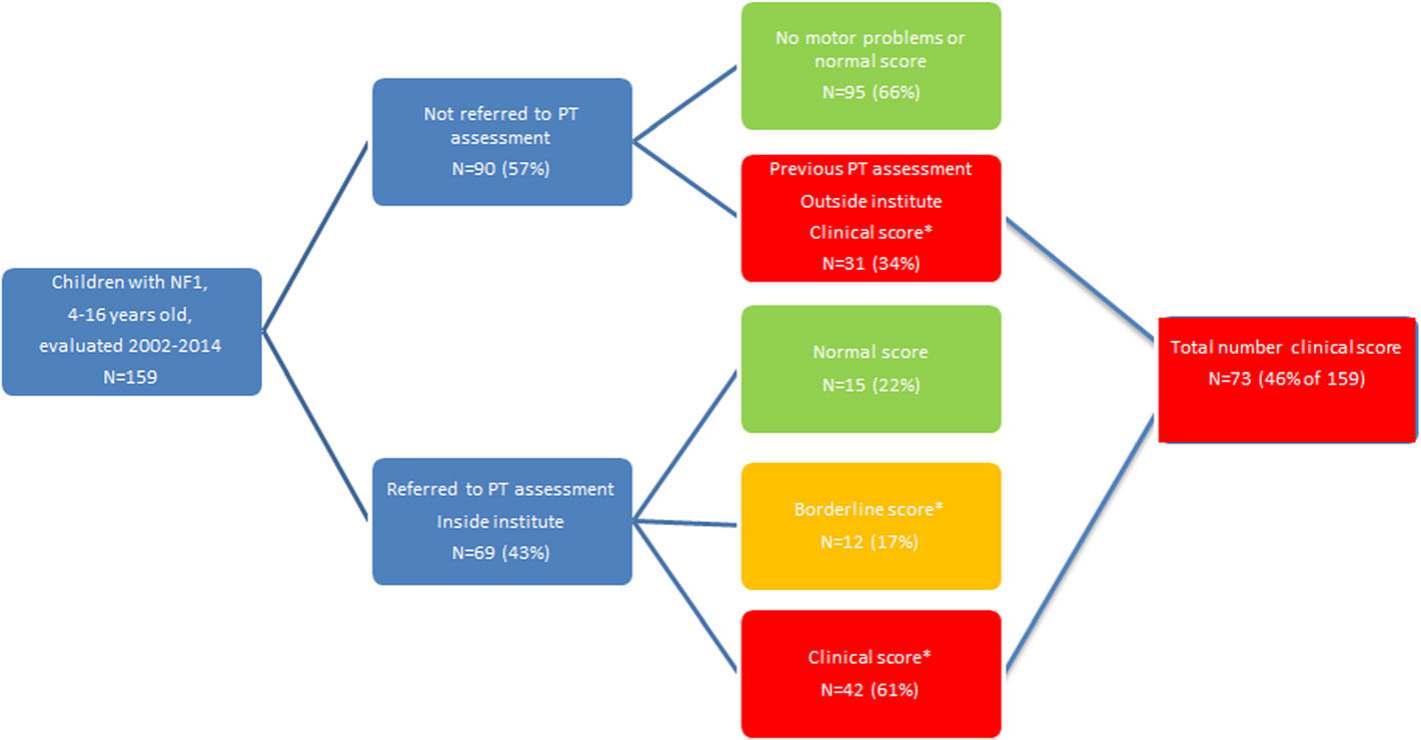
Flow chart of participants and outcomes. * M-ABC normal score >P15; borderline score P5 to <P15; clinical score <P5

The Kempenhaeghe Centre for Neurological Learning Disabilities (CNL) is an expertise centre for children with neurological learning disabilities such as NF1. At school age, a paediatric neurologist evaluates all patients at least once. Patients are offered additional evaluations by a neuropsychologist and a physiotherapist. Patients without any complaints about motor performance were not included in this study. Next to this, we did not re-evaluate the motor performance of patients who already had serious motor problems according to a recent evaluation by a physiotherapist using the Movement-ABC in a different institute. The selection process is depicted in Fig. 1. We used medical and psychological patient files from 2002 to 2014 of 4- to 16-year-old patients who met the National Institutes of Health (NIH) diagnostic criteria for NF1 [12] and who were evaluated by a physiotherapist using the Movement Assessment Battery for Children version 1 [13] or 2 [14] (M-ABC-1 or 2). Exclusion criteria were segmental NF1, symptomatic pathology of the CNS, deafness or severely impaired vision, pseudarthrosis, insufficient command of the Dutch language, or an IQ below the range covered by the Wechsler Intelligence Scale for Children, third edition, Dutch version (WISC-III-NL [15]; total IQ below 48).

Clinical data were registered by a paediatrician of Erasmus Medical Centre, Sophia Children’s Hospital during annual follow-up, by a paediatric neurologist from CNL and by psychologists from both centres. All children were evaluated according to a standardised protocol, routinely applied to all children with NF1 visiting the expertise centre. Familial or sporadic NF1 was determined from family history. In clinical assessments by the paediatric neurologist and the psychologist, the presence of neurologic, orthopaedic, or neuropsychiatric problems such as hypotonia, hypermobility, and scoliosis were recorded. Classifications of ADHD and ASD were based on neuropsychological assessment and on information from parents and teachers, using DSM-IV [16] criteria. Writing problems were reported by parents. Socio-economic status (SES) was derived from the zip code of the child’s home address using a standard Dutch classification system [17]. For the participating patients, a formal review and waiver was given by the medical ethical human research ethics committees of both the Erasmus Medical Centre and the CNL.

### Instruments

The Movement ABC, [13, 14] an instrument measuring the presence and severity of motor problems, is one of the most frequently and widely used standardised assessments of motor skills, also used in diagnosing developmental coordination disorder (DCD). To assess motor performance, the physiotherapist administered the M-ABC-1 [13] (2002-2010) or 2 [14] (2010-2014). The M-ABC assesses three components: manual dexterity, ball skills (catching and throwing), and balance (static and dynamic). The M-ABC is designed to identify and describe impairments in the motor performance of children and adolescents aged 4 to 12 (M-ABC-1) or 3 to 16 (M-ABC-2). The M-ABC-2 is an updated version of the M-ABC-1, not only the age range but also the sample size, have been expanded and more information on psychometric qualities has been acquired. Results on both tests are expressed in a score, with any child scoring below the 6th percentile of the normative sample being recorded as falling within the clinical range indicating serious movement difficulties. Scores from the 6^th^ to the 15^th^ percentile (approximately between 1.5 and 2.0 SD below average) are labelled as borderline, indicating that the child is at risk of motor problems. Above the 15th percentile, the child is unlikely to have movement difficulties. Additionally, the M-ABC-2 also provides norm-referenced standardised scores for the component and the total scores. The M-ABC-2 has good reliability (ICC = .95 to .98).

Intelligence was measured with the Wechsler Preschool and Primary Scale of Intelligence-Revised, Dutch version (first WPPSI-R [18], from 2010 WPPSI-III-NL [19]) or the WISC-III-NL [15]. These are intelligence tests for children, the first for those aged 2 years and 6 months to 7 years and 7 months, and the second for children aged 6 to 17 years. The tests consist of several subtests resulting in a Full-Scale IQ, Verbal IQ, and Performance IQ.

To assess emotional and behavioural problems, parents completed the Child Behavior Checklist (CBCL) using either the preschool version, the CBCL/1½–5 [20], or the school-aged version, CBCL/6–18 [21]. Scores were converted to *T* scores (mean 50, SD 10), with higher scores corresponding to more problems. Summed scores result in three broadband scales for Internalising, Externalising, and Total Problems. The Internalising Problems scale comprises anxious/depressed behaviour, withdrawn/depressed behaviour, and somatic complaints. The Externalising Problems scale comprises rule-breaking behaviour and aggressive behaviour. The Total Problems scale is a combination of both the Internalising and Externalising Problems scales, together with scales for Social Problems, Thought Problems, and Attention Problems. *T* scores between 59 and 62 fall within a borderline clinical range, whilst *T* scores of 63 and higher fall within the clinical range. All tests were administered in their Dutch versions, using Dutch normative samples.

### Statistical analysis

All data were analysed using SPSS, version 21 [22], and *R* [23]. Proportions of groups were compared using chi-square (*χ* ^*2*^) tests. Effect sizes were calculated using Cohen’s *d*, [24] when comparing the NF1 sample with the test manual normative sample, with .20 interpreted as a small effect size, .50 as medium, and .80 as large.

Since the common outcome for both versions of the M-ABC was the classification into three consecutive categories (normal, borderline, or clinical), ordinal logistic regression analysis was performed to find predictors of these three categories of motor outcome. For this, a two-phase strategy was followed. In phase 1, all separate variables from Table 1 were tested in univariable ordinal regression analyses with M-ABC classification as the dependent variable. Since this phase served as an initial, broad selection of potential predictors, *α* in phase 1 was set at .20 [25]. In phase 2, multivariable ordinal regression models were constructed for every block of variables from Tables 1 and 2, containing all significant variables from phase 1. Blocks were defined as demographics, neuropsychiatric problems, emotional and behavioural problems, and cognition. Variables shown to be significant contributors in the final models were regarded as the final predictors of M-ABC motor outcome (*α* in phase 2 was set at .05; stepwise backward elimination procedure).

**Table 1:** Characteristics of children with NF1

Variable	
Demographic characteristics	*n* = 69 Frequency (%)
Age	8.7 (4.1)^a^
Gender	
Male	40 (58)
Female	29 (42)
Type of education	
Regular education	48 (70)
Special education	21 (30)
Social economic status ^b^	0.34 (1.29)^a^
Mode of inheritance NF1	
De novo mutation	39 (57)
Familial mutation	29 (42)
Unknown	1 (1)
Neuropsychiatric problems	
Attention-deficit/hyperactivity disorder (ADHD)	
ADHD combined type	25 (36)
ADHD inattentive type	11 (16)
ADHD hyperactive/impulsive type	2 (3)
Total	38 (55)
Using stimulant medication	18 (26)
Autism spectrum disorder (PDD-NOS)	7 (10)
Neurologic and orthopaedic problems	
Hypotonia	14 (20)
Hypermobility (Beighton criteria)	13 (19)
Scoliosis	7 (10)

^a^Median (interquartile range)

^b^Average SES in 2010 = 0.17; higher scores indicate higher SES

**Table 2:** Scores and frequencies for emotional and behavioural problems, intelligence and motor performance

Domain	Number	Mean	SD^a^	BCR^b^ (%)	CR^b^ (%)	ES^c^
Parent-rated emotional and behavioural problems^d^						
Internalising problems	58	59	10	19	37	0.9^***^
Externalising problems	58	55	12	8	27	0.5^**^
Total problems	58	61	11	10	41	1.0^***^
Intelligence^e^						
Verbal IQ	68	92	15			0.5^***^
Performance IQ	68	88	14			0.8^***^
Total IQ	69	89	13			0.8^***^
Movement ABC-1 and 2 (*n* = 69)						
Classification normal^f^	15 (22%)					
Classification borderline^f^	12 (17%)					
Classification clinical^f^	42 (61%)					
Movement ABC-2^g^ (*n* = 34)						
Manual dexterity	34	5.8	3.3			1.3^***^
Ball skills	34	6.7	3.6			1.0^***^
Balance	33	5.7	3.0			1.4^***^
Total	34	4.8	3.2			1.7^***^

^a^ *SD* Standard deviation

^b^ *BCR/CR* Percentage of scores in borderline clinical range/clinical range

^c^ *ES* effect size (Cohen’s *d*); Significance compared to normative sample ^**^ *p* < .01; ^***^ *p* < .001

^d^ *T* scores (population mean = 50; SD = 10; higher scores reflect more problems)

^e^IQ scores (population mean = 100; SD = 15; higher scores reflect better performance)

^f^M-ABC normal score >P15; borderline score P5 to <P15; clinical score <P5)

^g^Standard-scores (population mean = 10; SD = 3; higher scores reflect better performance)

## Results

### Patient characteristics

From 2002 to 2014, 159 children with NF1 aged 4 to 16 years old visited the expertise centre. Ninety (57% of 159; 46 girls and 44 boys) were not referred to the physiotherapist, of which 31 (34% of 90) had a previous assessment outside our institute, indicating serious motor problems, according to M-ABC scores in the clinical range. Of the other 59 (66% of 90), parents and children did not have any complaints about motor performance before or during their visit to our institute. In the flow chart of Fig. 1, we have visualised the distribution of the sample. For this study, 69 children (43% of 159) with NF1 were included for PT evaluation in our institute, 29 girls, and 40 boys. This group is indicated in the box ‘Referred to PT assessment’ in Fig. 1. Ages ranged from 4 years to 15 years and 11 months, with a median age of 8 years and 8 months (IQR = 4 years and 1 month) (Table 1). Sixty-seven children were right handed. Eighteen out of 38 of the children with a DSM-IV-TR classification of ADHD (47%) used stimulant medication. Seven children had an ASD classification, all of them with a comorbid ADHD classification. Intelligence, emotional and behavioural problems, and standard scores of the M-ABC-2 are presented in Table 2.

Twenty-four children (41%) had emotional and behavioural problem scores within the clinical range, with large effect sizes for internalising problems and medium effect sizes for externalising problems. Parents of 11 children (16%) did not return CBCLs. These children were left out of analyses using CBCL scores as predictors. Compared to the normative sample, the distribution of intelligence scores was shifted approximately one SD to the left, and total IQ scores ranged from 58 to 123. Effect sizes were large for performance IQ and medium for verbal IQ compared to the normative population. Effect sizes for all motor scales were large.

### Motor problems

**Fig. 2:**
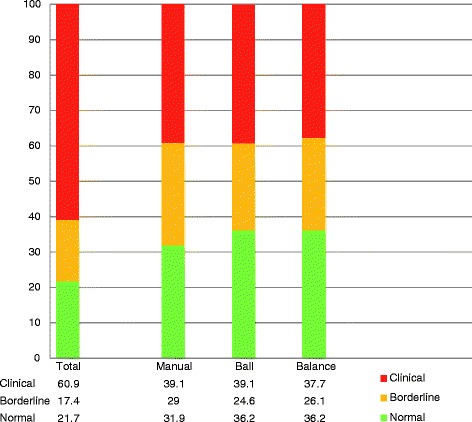
Classification of motor problems based on Movement ABC percentile scores (*n* = 69). *Clinical*: percentage of children with movement difficulty- scores below 6th percentile. *Borderline*: percentage of children with scores from 6th to 15th percentile. *Normal*: percentage of children with no movement difficulty scores above 15th percentile

Thirty-five of 69 children (51%) were assessed with the M-ABC version 1, 34 (49%) with version 2. The comparison between children tested with these two versions showed no significant differences in the distribution of scores between the percentile classification categories for the total scores (*χ* ^*2*^ (2) = 3.08, *p* = .21), nor for distributions of Hand, Ball, or Balance scale scores. For the purpose of ordinal regression analyses, both groups were combined. Figure 2 presents the distribution of the classifications in all motor scales. Overall, 42 (61%) children with NF1 scored within the clinical range (below 6th percentile) of the M-ABC.

In ordinal regression analysis, age was found not to be a significant predictor of motor outcome. The proportion of children scoring in the ‘borderline’ or ‘clinical’ range of the M-ABC was 67% of children from 4 to 6 years old, 82% of children from 7 to 11 years old, and 79% of adolescents from 12 to 16 years old.

In univariable ordinal regression analysis (Table 3; phase 1 with *α* set at .20), a higher probability of borderline or clinical motor problems was predicted by type of education, classifications of ADHD or ASD, hypermobility, Performance IQ, Total IQ, and CBCL Internalising, Externalising, and Total Problems. In all univariable models, the test of parallel lines failed to reach significance, meaning that effects of all separate variables were the same for normal versus borderline and borderline versus clinical scores.

**Table 3:** Univariable ordinal logistic regression with separate variables predicting motor outcome (Movement ABC total scores; *n* = 69)

Variable	Number	B (SE)	95% CI of odds ratio			Wald	*R* ^2^	*p*
			Lower	OR	Upper			
Age	69	0.08 (0.09)	0.77	0.93	1.12	4.31	.01	.429
Gender	69	0.56 (0.49)	0.22	0.57	1.49	1.31	.02	.253
Type of education	69	0.84 (0.58)	0.14	0.43	1.35	2.08	.04	.135^#^
Social economic status	69	−0.10 (0.19)	0.77	1.10	1.59	0.29	.01	.595
Mode of inheritance	69	−0.42 (0.49)	0.59	1.53	3.95	0.76	.01	.383
ADHD	69	1.01 (0.49)	0.14	0.36	0.96	4.22	.07	.038^*^
Using stimulant medication	69	−0.35 (0.54)	0.49	1.41	4.05	0.41	.01	.523
Autism spectrum disorder^a^	69	–	–	NA	–	–	.12	.035^**^
Hypotonia	69	0.28 (0.60)	0.23	0.76	2.47	0.21	<.01	.644
Hypermobility	69	0.98 (0.70)	0.10	0.38	1.49	1.94	.04	.140^#^
Scoliosis	69	0.25 (0.79)	0.28	1.28	5.97	0.10	<.01	.755
Writing problems at school	69	−0.20 (0.50)	0.46	1.22	3.25	0.16	<.01	.694
CBCL Internalising problems	58	−0.04 (0.03)	0.99	1.04	1.09	2.23	.04	.134^#^
CBCL Externalising problems	58	−0.04 (0.02)	0.10	1.04	1.09	3.30	.07	.063^#^
CBCL Total problems	58	−0.05 (0.03)	1.00	1.05	1.10	3.65	.08	.051^#^
Verbal IQ	68	0.01 (0.02)	0.96	0.99	1.02	0.41	.01	.519
Performance IQ	68	0.03 (0.02)	0.94	0.97	1.01	2.43	.04	.115^#^
Total IQ	69	0.03 (0.02)	0.94	0.97	1.01	2.10	.04	.141^#^

*R* ^*2*^ Nagelkerke pseudo *R* ^2^

*p* values of likelihood ratio chi-square; ^#^ *p* < .20; ^*^ *p* < .05; ^**^ *p* < .01

^a^As there were no cases in cells with normal M-ABC-scores for children with an ASD classification, the estimate was minus infinity

In multivariable ordinal regression analyses (Table 4; phase 2 with *α* set at .05), single variables within one block (type of education and hypermobility) had *p* values above *α* = .05 and so could not be used in multivariable models. In three blocks, final models yielded a limited amount of significant predictors. Since all seven children with an ASD classification scored within the clinical range, the odds ratio of having borderline or clinical M-ABC scores could not be calculated and so ASD was left out of multivariable analyses. Also, the multivariable ordinal regression of ADHD and ASD could not be performed because all seven children with ASD classifications also had an ADHD classification. We compared the distribution of the M-ABC classification between the groups without ADHD or ASD versus the group with only ADHD versus the group with both ADHD and ASD using a chi-squared test. This distribution did not differ significantly (*χ* ^*2*^ (4, *N* = 69) = 7.53, *p* = .11), indicating that all three groups contributed independently to the distribution of motor problems.

**Table 4:** Multivariable backward ordinal logistic regression with variables from separate blocks predicting motor outcome (Movement ABC total classification; *n* = 69)

Variables	Number	B (SE)	95% CI of OR			Wald	*R* ^2^	*p* value
			Lower	OR	Upper			
Neuropsychiatric problems								
ADHD	69	1.01 (0.49)	0.14	0.36	0.96	4.22	.07	.038^*^
Autism spectrum disorder^a^	69	–	–	NA	–	–	.12	.035^*^
Emotional and behavioural problems								
Model 1	58						.07	.168
Internalising problems		−0.01 (0.03)	0.95	1.01	1.08	0.10		.757
Externalising problems		−0.04 (0.03)	0.98	1.04	1.10	1.44		.235
Model 2	58							
Externalising problems		−0.04 (0.02)	0.10	1.04	1.09	3.30	.07	.063
Intelligence								
Model 1	68						.04	.289
Performance IQ		0.03 (0.03)	0.91	0.97	1.04	0.71		.401
Total IQ		0.001 (0.03)	0.94	1.00	1.07	0.001		.973
Model 2	68							
Performance IQ		0.03 (0.02)	0.94	0.97	1.01	2.43	.04	.115

*OR* odds ratio, *NA* not applicable, *R* ^*2*^ Nagelkerke pseudo *R* ^2^

*p* values of likelihood ratio chi-square; ^*^ *p* < .05

^a^As there were nog cases in cells with normal M-ABC-scores for children with an ASD classification, the estimate was minus infinity

**Fig. 3:**
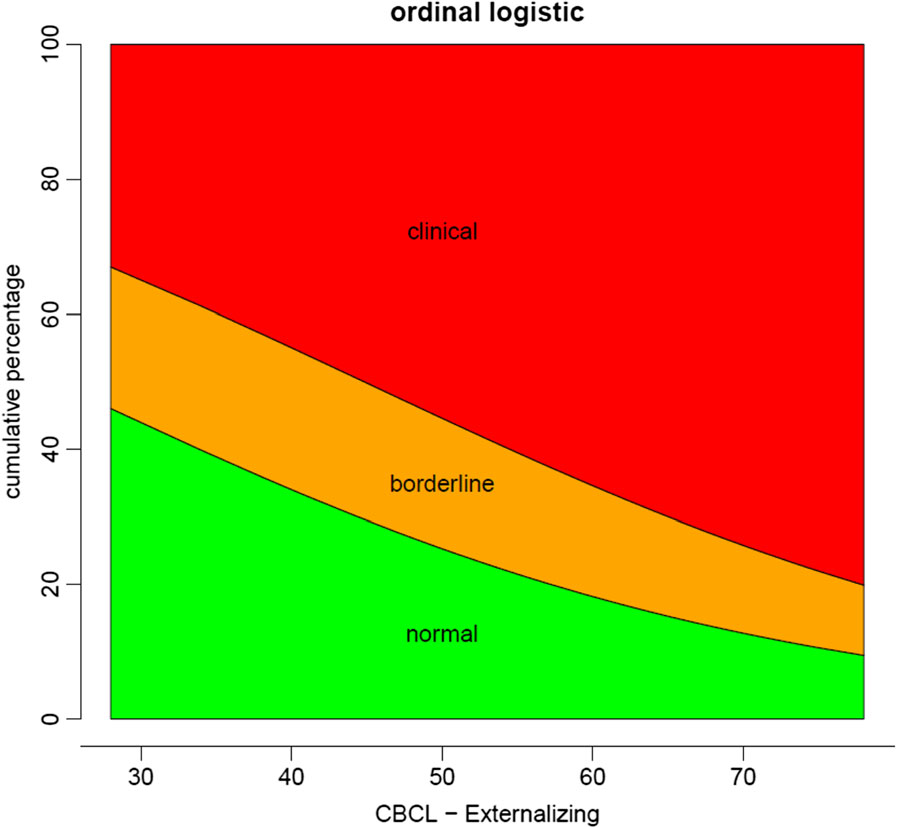
Relationship between cumulative percentages of classification of total motor scores and scores on CBCL Externalising problems scale

The Externalising Problems scale was approaching significance as a predictor of motor outcome (*p* = .063). With low scores for Externalising Problems, the probability of a clinical score on the M-ABC was low. Children without externalising problems on the CBCL only had a 23% chance of a clinical score on the M-ABC, whilst children with externalising problems scores in the clinical range had an 81% chance, as is shown in Fig. 3. Finally, intelligence (i.e. Performance IQ) was not found to be significantly associated with total motor problems.

Exploratory univariable linear regression analyses, with motor outcome on the M-ABC-2 as a continuous dependent variable (*n* = 34), found significant associations with independent variables: Internalising Problems scale (*F* (1,26) = 5.21; *p* = .031; *R* ^*2*^ = .17; *β* = −.13); Externalising Problems scale (*F* (1,26) = 6.99; *p* = .014; *R* ^*2*^ = .21; *β* = -.12); and Total Problems scale (*F* (1,26) = 6.15; *p* = .020; *R* ^*2*^ = .19; *β* = −.13), again indicating that an increase in emotional and behavioural problems is associated with a decrease in motor proficiency. Residuals for these regressions were normally distributed.

## Discussion

Our study shows that motor problems frequently occur in our group of children with NF1: 61% of these 69 children have serious motor problems and another 17% score within the borderline range. In the part of our cohort not evaluated in the expertise centre (*n* = 90), 31 were already identified as having motor problems, resulting in an overall 46% (73/159) with serious motor problems. The distribution of these groups and outcomes is visualised in Fig. 1 in the box with ‘Total number clinical score’. Previous studies using broad motor test batteries found smaller or comparable proportions. One study in a comparable age range found 54% (14 out of 26 children) scoring between one and two standard deviations below average and another 27% (7/26) scored below 2 SD [6]. When comparing studies, one should realise that the cut-offs of the P5 and the P15 correspond to *z* scores of 1.65 and 1.04 below average in the standard normal distribution.

Next to ADHD [26] and ASD symptoms [27], motor problems seem to be among the most common comorbid developmental problems of children with NF1. We found motor problems in a broad range of domains, comparable to the problems found in DCD [8].

In our attempt to find predictors of motor outcome, we did not find a significant contribution of demographic characteristics such as age, gender, or SES. A previous comparable study in a smaller sample did not find effects for age or gender either [6]. We also did not find associations with neurological and orthopaedic problems such as hypotonia, hypermobility, or scoliosis. Given the broad variability in these characteristics within our population (Table 1), we think our study population had sufficient power to detect potential associations if they existed. There was a limited association between (performance) intelligence and motor performance. Previous research [4] found that motor coordination and motor speed contributed to the performance on some subtests of the WISC. However, in our study, we used a broader motor test battery such as the M-ABC and children were found to have serious motor problems in general, regardless of their overall intelligence. Since a previous study [9] found that poorer balance skills were associated with a reduced perceptual reasoning index, we performed an additional univariable ordinal logistic regression to specifically find out whether balance skills on the M-ABC were associated with performance IQ. Only a weak association was found with an odds ratio of 0.97 (95% CI, 0.93 to 1.00), Wald *χ* ^2^ (1) = 3.774, *p* = .052). Whether this finding is a reflection of an abnormal neurodevelopmental process, underlying these abilities in children with NF1, may be a subject for future research.

Recent studies do provide evidence for a relation between motor experience and cognitive development in the first 3 years of life when at the same time this relation becomes less clear in older children [28]. The fact that we did not find a significant effect of age on motor performance may presumably be caused by the fact we included children from 4 to 16 years old.

Externalising behavioural problems might be associated with motor outcome. This association was found to be significant in additional explorative analyses with standard scores of the children tested with the M-ABC-2. Also, ADHD was a significant predictor of motor outcome, and all children with an ASD classification had severe motor problems. Previous studies also found that motor problems often occur in children with emotional, behavioural, and pervasive developmental disorders [29, 30]. The co-occurrence of motor and behavioural problems could be an indicator of a more severe neurologic phenotype [31]. It is, however, unclear what the direction of the association between behavioural and motor problems is. Longitudinal and treatment studies could elucidate this issue. Neuropsychiatric and motor problems have a large impact on participation in daily life, even more so when these problems occur simultaneously.

### Limitations

Although NF1 is relatively rare, we succeeded in gathering data on the motor performance of 69 children over a 12-year period. However, our sample size is still small considering the number of variables incorporated into the regression analyses of this study. For this reason, there is a risk of overfitting, and care should be taken when drawing conclusions regarding the predictive value of variables. To avoid unnecessary assessments, we did not evaluate the motor performance of children who recently had such an assessment. In addition, since the assessment of motor performance was on a voluntary basis, children without any motor complaints were not required to visit our physiotherapist. For these reasons, we cannot exclude selection bias. We tried to correct for this bias by calculating the total amount of children scoring in the clinical range (Fig. 1).

The cross-sectional design limits interpretations regarding the effect of age on motor performance. Probably, longitudinal research will be able to express this relationship in a more decisive way.

During the time period of this study, there was a move by physiotherapists in the Netherlands from using the first version of the Movement-ABC to the second version. For this reason, we were dependent on the categorical classification of motor problems as a primary outcome measure. This is a consequence of continuous sampling over a long period of time. One should be careful when combining data from both tests since the M-ABC-2 is an elaboration of the M-ABC-1, resulting in differences between both instruments [32]. Because the age range of the M-ABC was the starting point of this study, we used the two age-appropriate versions of the Wechsler scales and of the CBCL. Although the correlation between both versions is high, [19, 20] future research in larger groups could benefit from the selection of smaller age ranges.

For this study, we collected data from medical records. This resulted in missing information (as is shown in Table 2), particularly regarding emotional and behavioural problems, most likely because some parents failed to return questionnaires. Since all children were assessed using a standardised protocol, other data are relatively complete.

The proportion of children with ADHD symptoms is comparable to that in other studies, [26] but the percentage of children with ASD symptomatology in our study (10%) is somewhat lower than former prevalence estimates (21–40%) [33]. In the group with ASD, all children appeared to have severe motor problems. Although this may suggest clinical relevance, we interpret this observation with care, due to the small sample size.

### Clinical implications and recommendations

Developmental motor problems are frequently overlooked in clinical practice, yet they can have a considerable impact on children’s lives [34]. Using a broad motor assessment in a large cohort of children with NF1, we showed a high prevalence of serious motor problems. These problems seem to be independent of age or intelligence. When children with NF1 show serious motor problems, the diagnosis of DCD might be considered as a comorbid problem. This is especially important in helping to recognise the impact of motor problems on daily life and in allocating the correct treatment. Although the DSM-IV-TR [16] states that in DCD, ‘the disorder is not due to a general medical condition’, to our opinion NF1 does not have to be regarded as such. DCD could be used in practice as a descriptive diagnosis stressing the impact of motor problems on daily life.

Concerning participation in daily life, children with NF1 often experience problems with writing [4, 35]. It is important to find out whether people with NF1 experience further such difficulties in daily functioning such as in activities of daily living, play, sports, or with driving. This is of great importance since a decrease in participation could not only affect the practice of motor skills but also the development of social skills and quality of life in general.

Assessment and treatment of motor problems in NF1, especially in children with behavioural and social problems, should be considered at a young age, using a broad motor assessment battery. Early motor intervention can have a beneficial effect on behavioural problems, as is indicated by a study showing that in ADHD, [36] motor-affected children receiving physiotherapy presented less frequently with comorbid emotional and behavioural problems. The impact of physiotherapy and psychological therapy on motor functioning, motor participation, and emotional and behavioural problems in children with both NF1 and motor problems is unknown. However, considering the larger potential for plasticity at a young age, referral to both a physiotherapist and a psychologist could be considered at a young age in children with NF1.

## Conclusions

More than half of the children with NF1 in this sample had severe motor problems. These problems seem to be independent of age or intelligence. Next to ADHD and ASD, motor problems are among the most frequent comorbid developmental problems in children with NF1. In this study, ADHD and ASD symptomatology, and externalising behavioural problems are associated with motor problems. The combination of both motor and behavioural problems might result in a more severe phenotype of NF1. Because of their impact on participation in daily life, motor problems need to be specifically addressed in diagnosis, follow-up, and treatment of children with NF1.

## Abbreviations

ADHD: Attention-deficit/hyperactivity disorder
ASD: Autism spectrum disorder
CBCL: Child Behavior Checklist
CNL: Kempenhaeghe Centre for Neurological Learning Disabilities
DCD: Developmental coordination disorder
DSM-IV: Diagnostic and statistical manual of mental disorders
IQ: Intelligence quotient
M-ABC-1 or 2: Movement Assessment Battery for Children version 1 or 2
NF1: Neurofibromatosis type 1
SES: Socio-economic status
WISC-III-NL: Wechsler Intelligence Scale for Children, third version for the Netherlands
WPPSI-III-R: Wechsler Preschool and Primary Scale of Intelligence—Third version for the Netherlands
